# Bureau of Surgery, I. H. A.

**Published:** 1889-01

**Authors:** 


### BUREAU OF SURGERY, I. H. A.

The Homœopathic Physician of August, 1888, says of this part of our work: "This is an important bureau in the I. H. A., as it endeavors to show how the law of similars aids the surgeon; how it removes in many cases the necessity for the use of the knife, and also how, in cases where operations must be performed it saves the patient from dangerous sequelæ. It is an important work, and should not be slighted. Under homœopathic medication many surgical operations are rendered needless, and many operations are made possible, when under allopathic treatment death must surely follow."

The whole paragraph quoted above may serve as an excellent introduction to the surgical work of the I. H. A. for 1889.

It shows how the work appropriated to this bureau is not confined to those who operate more or less, but that any one who tends a case of cancer, tumor, syphilis, gonorrhœa, ulcer, aneurism, osteitis, caries, necrosis, gangrene, cystitis, enlarged prostate, hemorrhoids, anthrax, ascites, fissure of anus, condylomata, warts, corns, coccyodynia, acne, comedones, impotence, lockjaw, lupus, orchitis, vaginismus, enlarged tonsils, white swelling—as well as many other affections of the eye, ear, teeth, nose, and male and female genito-urinary organs, rectum, etc., etc., is practicing homœopathic surgery, and will be able to report "how it removes in many cases the necessity for the use of the knife."

There is not a member of the I. H. A. who is not practicing such excellent surgery as this, and every one should make some report of it. Look over the best allopathic surgical text-books and see how many surgical cases you are treating, and how much better you are doing it than the allopaths, and give us your experience for the benefit of the world. Bear it in mind from this time on, and you will surely observe something worth recording. Have it ready by June 1st, and send to any of the following members of the Bureau.

E. Carlton, M. D., 58 W. Ninth St., New York.
Geo. H. Clark, M. D., W. Walnut Lane, Gtn., Phila.
C. H. Lawton, M. D., Wilmington, Del.
T. M. Dillingham, M. D., 134 Boylston St., Boston.
J. H. Payne, M. D., 415 Columbus Ave., Boston.
F. L. McIntosh, M. D., Melrose, Mass.
A. McNeil, M. D., 220 Turk St., San Francisco, Cal.
James B. Bell, 178 Commonwealth Ave., Boston,
*Chairman.*

